# Recent Advances in Liquid Chromatography–Mass Spectrometry (LC–MS) Applications in Biological and Applied Sciences

**DOI:** 10.1002/ansa.70024

**Published:** 2025-06-27

**Authors:** Samyah Alanazi

**Affiliations:** ^1^ Clinical Laboratory Sciences Department College of Applied Medical Sciences King Saud University Riyadh Saudi Arabia

**Keywords:** food analysis | life science | liquid chromatography–mass spectrometry (LC–MS) | omics | pharmaceuticals

## Abstract

Liquid chromatography–mass spectrometry (LC–MS) is a highly sophisticated analytical technique that has become indispensable for sample analysis across various scientific domains. In recent years, LC–MS has emerged as a cornerstone technology in comparative replicate sample analysis, particularly in the fields of proteomics, lipidomics and metabolomics. This review provides a comprehensive and in‐depth exploration of LC–MS applications in biological, and applied sciences from the key aspects include (1) a historical perspective on LC–MS development and advancements; (2) its critical role in forensic investigations and environmental science; (3) its applications in food safety, quality control and pharmaceutical analysis and (4) its significance in the examination of pharmaceuticals and biological specimens. By presenting a broad and integrated understanding of LC–MS, this review underscores its transformative impact on life sciences and its expanding role in scientific research and innovation.

## 1 Introduction

Liquid chromatography coupled with mass spectrometry (LC–MS) is a well‐established analytical technique known for its high accuracy and time efficiency in metabolite analysis. Over time, it has evolved to play a crucial role in biological metabolite research [1]. LC–MS‐based techniques are widely regarded as essential tools in metabolomics studies [2], particularly for monitoring metabolites in various research fields, including plant, human and animal sciences [2]. Due to its high sensitivity, specificity and rapid data acquisition, LC–MS is well suited for detecting a broad spectrum of nonvolatile hydrophobic and hydrophilic metabolites [3]. The integration of novel ultra‐high‐pressure techniques with highly efficient columns has further enhanced LC–MS, enabling the study of complex and less abundant bio‐transformed metabolites [2]. Recently, concerns have arisen regarding the presence of veterinary drug residues in food products derived from animals [4]. LC–MS serves as a robust analytical approach for monitoring and quantifying these residues, ensuring that animal‐derived products meet safety standards before entering the food supply chain [4]. Through LC–MS methodologies, the food industry can ensure compliance with regulatory requirements while providing consumers with confidence in food safety [3]. LC–MS plays an extensive role in biological sciences [5], particularly in pharmaceutical research, where it is applied across various stages of drug discovery and development [6]. This powerful technique facilitates the investigation of complex biological systems, aiding in the identification of disease mechanisms and the rapid discovery of new therapeutic agents [5]. Owing to its exceptional ability to classify, identify and quantify compounds with unparalleled sensitivity and accuracy, LC–MS is a preferred tool in analytical chemistry [7]. Recent advancements in ‘‐omics’ techniques have further elevated the importance of LC–MS in systems biology, allowing for the large‐scale analysis of metabolites with unprecedented detail [8]. LC–MS has solidified its presence across multiple disciplines, including pharmacology, proteomics, metabolomics, environmental research and food analysis [8].

The versatility and robustness of LC–MS make it an indispensable instrument for researchers in academia, industry and regulatory agencies. This review aims to provide a comprehensive exploration of LC–MS applications, highlighting its transformative impact across multiple scientific fields. Additionally, the review examines the pivotal role of LC–MS in forensic investigations, environmental monitoring, food safety and biopharmaceutical research. By the end of this review, readers will gain a thorough understanding of LC–MS's multifaceted applications and its significance in advancing biological and life sciences.

## 2 Historical Development of LC–MS

**FIGURE 1:**
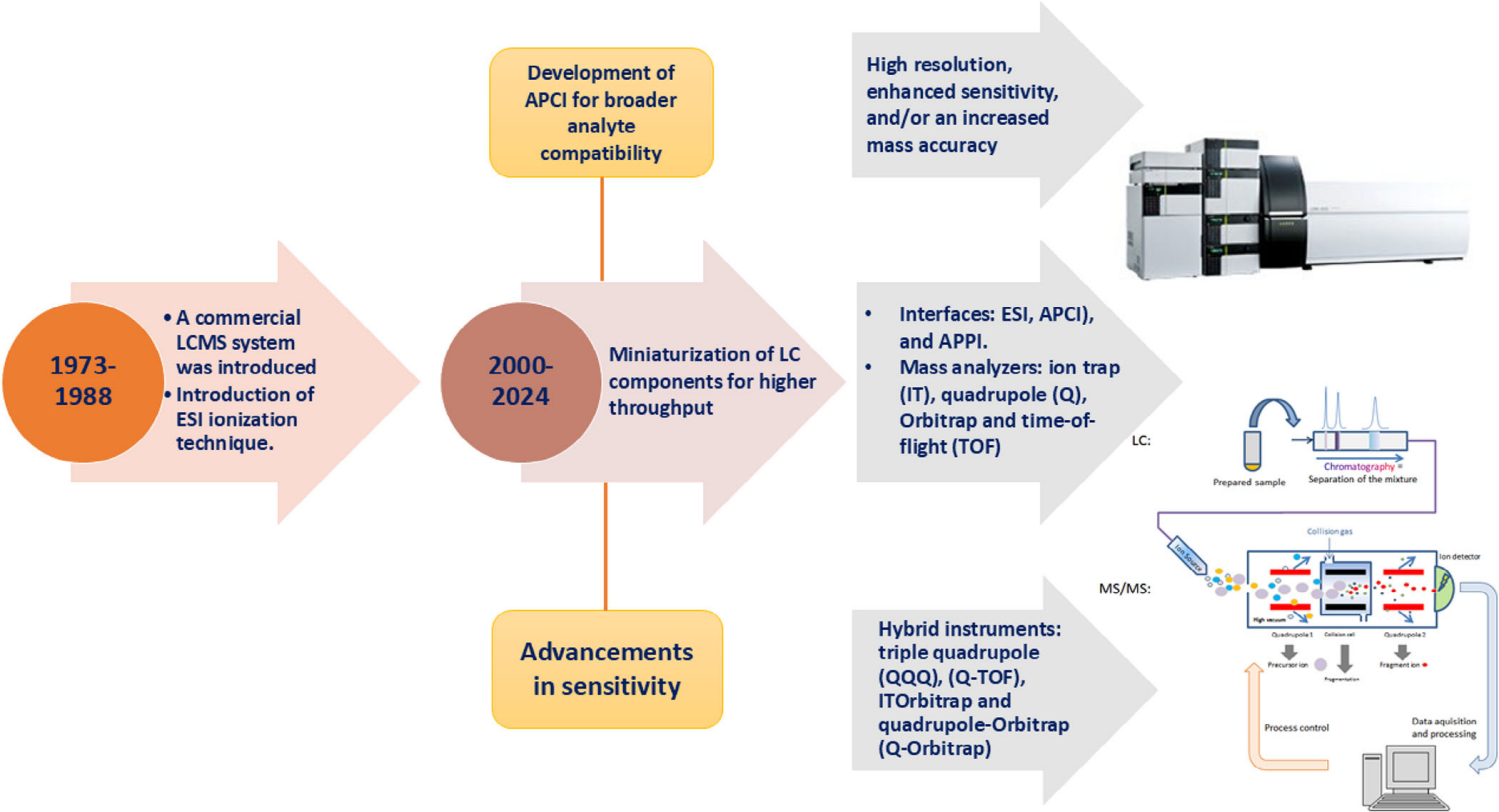
Progress in LC–MS development in the last four decades. APCI, atmospheric pressure chemical ionization; APPI, atmospheric pressure photoionization; ESI, electrospray ionization; LC–MS, liquid chromatography–mass spectrometry.

The development of LC–MS has had a profound impact on the biological and analytical sciences, ushering in a new era of advanced analytical methodologies [9]. This technique stands as a testament to the tremendous advancements in analytical methodologies. The historical development of LC–MS is marked by groundbreaking innovations, critical turning points and its enduring impact on scientific discovery (Figure 1).

The integration of LC–MS was first conceptualized in the mid‐20th century, as the analytical chemistry community sought to develop a versatile tool for complex sample analysis [10]. Early breakthroughs in both fields laid the foundation for the development of LC–MS, merging the separation capabilities of LC with the structural elucidation power of MS. This integration led to a revolutionary shift in analytical chemistry, providing researchers with an unparalleled ability to study intricate mixtures, including pharmaceuticals, proteins and biological matrices [10]. One of the most significant milestones in the development of LC–MS occurred in the 1970s, when the first commercial LC–MS system was introduced. This marked the beginning of a new era for analytical techniques, allowing scientists to combine the advantages of both LC and MS for real‐time, accurate, and high‐resolution analysis [11]. This early version of the LC–MS system utilized quadrupole mass spectrometers, which provided good sensitivity and resolution for basic applications. Throughout the 1980s and 1990s, the technology continued to evolve with the introduction of new ionization techniques that expanded LC–MS's capabilities. Among the most important were electrospray ionization (ESI) and atmospheric pressure chemical ionization (APCI), both of which significantly enhanced sensitivity and widened the range of analytes that could be detected [6]. These techniques enabled the analysis of large, polar biomolecules such as proteins, peptides and nucleic acids, marking a turning point for biomolecular research. To further improve sensitivity and metabolite quantification, advanced applications such as twin derivatization–based LC–MS (TD–LC–MS) and chemical isotope labelling (CIL)‐based LC–tandem mass spectrometry (MS/MS) were introduced [2]. The addition of MS/MS also enabled deeper structural analysis of molecules, facilitating the study of metabolites, proteins and pharmaceuticals with greater precision. The contributions of LC–MS to the fields of analytical chemistry and life sciences are immeasurable. It has revolutionized the way complex biological, environmental, and pharmaceutical samples are analysed, providing researchers with the ability to perform high‐throughput analyses with precision [12]. Today, LC–MS is indispensable in numerous disciplines, including pharmaceuticals, environmental sciences, and food safety [2, 13, 14]. Its ability to detect a wide range of analytes at low concentrations has made it a critical tool for everything from drug development to biomarker discovery and toxicological analysis.

## 3 Advancements in LC–MS Instrumentation

**FIGURE 2:**
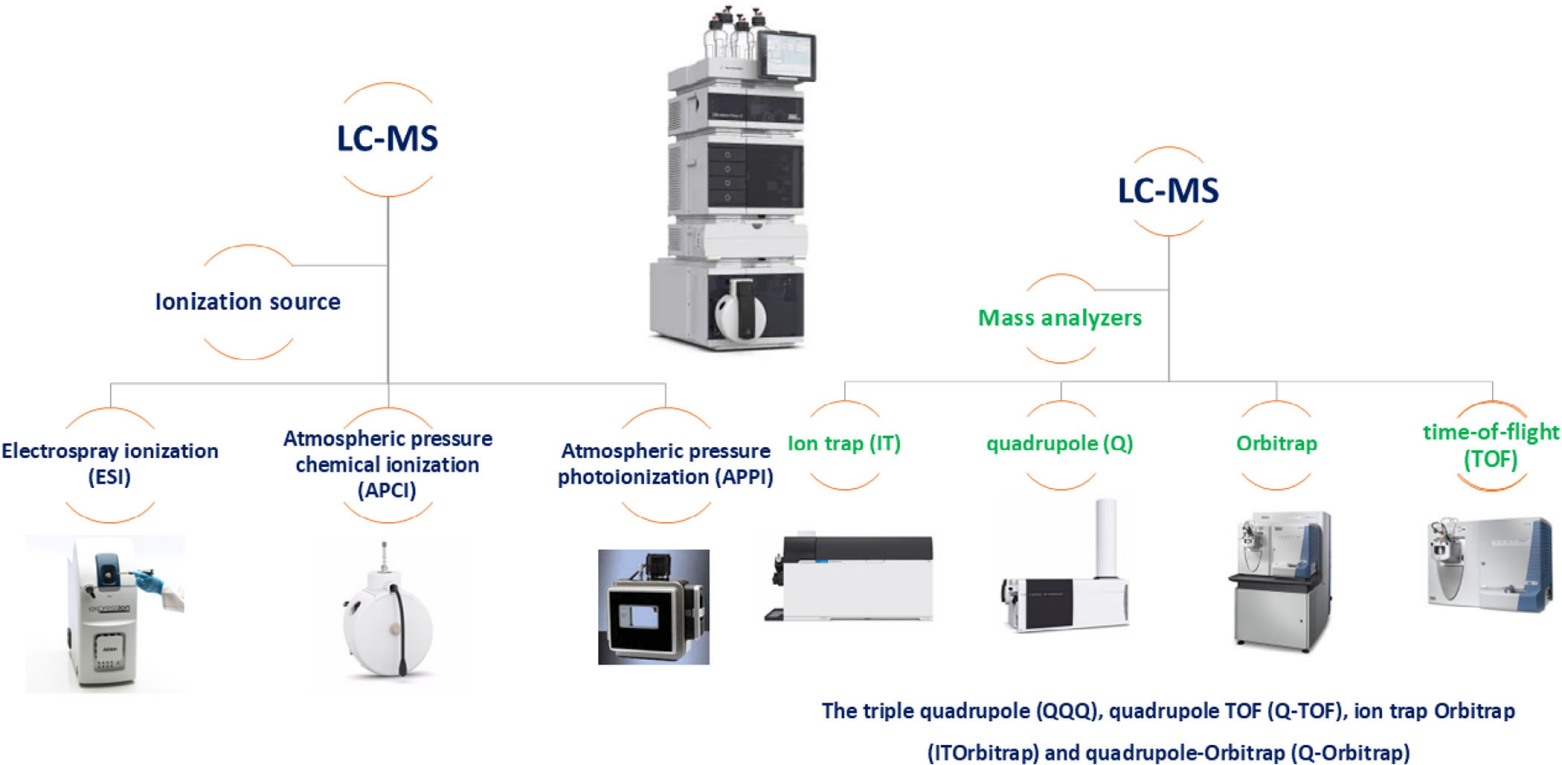
Advancement in LC–MS ionization sources and MS‐analysers. LC–MS, liquid chromatography–mass spectrometry.

The continuous improvement of instrumentation is the key to LC–MS's success [15]. The advancement of LC and MS components has been instrumental in the evolution of LC–MS technology [15]. LC systems have evolved from basic manual pumps and columns to sophisticated automated systems that provide precise control over chromatographic separations [6]. Miniaturization of LC components has led to higher throughput and reduced sample requirements, making LC–MS more efficient and practical [6]. The development of ionization sources has also had a profound impact on LC–MS performance (Figure 2) [6]. Techniques such as ESI, APCI and atmospheric pressure photoionization (APPI) have facilitated the analysis of pharmaceutical compounds, including nonvolatile and polar or less polar molecules with lower molecular weights [6, 16]. Mass analysers have also undergone significant improvements. Commonly used analysers in pharmaceutical analysis include ion traps (ITs), quadrupoles (Q), Orbitrap and time‐of‐flight (TOF) instruments, as well as hybrid systems offering high resolution, enhanced sensitivity and superior mass accuracy across a wide dynamic range [17]. Among these, triple quadrupole (QQQ), quadrupole TOF (Q‐TOF), ion trap‐Orbitrap (IT‐Orbitrap) and quadrupole‐Orbitrap (Q‐Orbitrap) remain particularly popular [6]. LC–MS systems can operate in full‐scan mode for untargeted analysis or in targeted acquisition modes such as single‐ion monitoring (SIM) and extracted ion chromatogram (EIC) for precise compound detection. MS/MS, which enables sequential mass analysis to investigate compound fragmentation behaviour, has further enhanced LC–MS capabilities in pharmaceutical applications [18, 19].

A significant revolution in LC–MS technology has been the dramatic increase in sensitivity and resolution [20]. Improved ion optics, mass analysers, and detectors have enabled LC–MS systems to detect analytes at picogram and femtogram levels, facilitating trace molecule identification in complex matrices. This increased sensitivity has significantly benefited various applications, including drug metabolite analysis and environmental contaminant detection [21].

## 4 Impact of LC–MS on Life Sciences

LC–MS has revolutionized life sciences by providing highly sensitive, selective and high‐throughput analytical capabilities. It has become an indispensable tool in diverse biological research fields, enabling a deeper understanding of biochemical pathways, disease mechanisms and biomolecular interactions [22]. One of its most significant contributions is in omics‐based sciences, where it has enhanced the ability to study proteins, metabolites, and nucleic acids with clear details. Proteomics, metabolomics and genomics have particularly benefited from LC–MS advancements, allowing for the precise quantification of biomolecules, structural characterization and the identification of novel biomarkers for disease diagnosis and prognosis [23].

In drug discovery and development, LC–MS has drastically improved the efficiency and accuracy of pharmaceutical research. It facilitates the identification of lead compounds, assessment of pharmacokinetics (PKs), metabolic profiling, and drug metabolism and pharmacokinetics (DMPK) studies [24]. By enabling rapid and comprehensive screening of drug candidates, LC–MS has accelerated the development of personalized medicine, paving the way for patient‐specific therapeutic strategies based on metabolic and proteomic profiling [25]. Moreover, advancements in ultra‐high‐performance liquid chromatography–mass spectrometry (UHPLC–MS) have further expanded the capabilities of pharmaceutical and biomedical research. UHPLC–MS offers significantly reduced analysis times (2–5 min per sample), allowing for high‐throughput screening, combinatorial synthesis monitoring and real‐time metabolic studies. This makes it particularly attractive for applications in continuous drug development pipelines, pharmacovigilance and toxicology studies, where large datasets need to be processed efficiently without compromising accuracy [6]. The ability of LC–MS to operate in 24/7 routine workflows enhances the reliability of research outcomes while ensuring faster drug development cycles. Beyond pharmaceuticals, LC–MS is increasingly applied in clinical diagnostics, environmental monitoring, food safety and forensic science. It plays a key role in detecting biomarkers of disease, identifying contaminants in food and water and analysing forensic evidence with high specificity. With continuous advancements in high‐resolution mass spectrometry (HRMS), ion mobility spectrometry (IMS) and machine learning (ML)‐based data analysis, LC–MS is expected to further transform life sciences by providing deeper insights into complex biological and chemical systems [26].

## 5 Applications for LC–MS in Forensics

**FIGURE 3:**
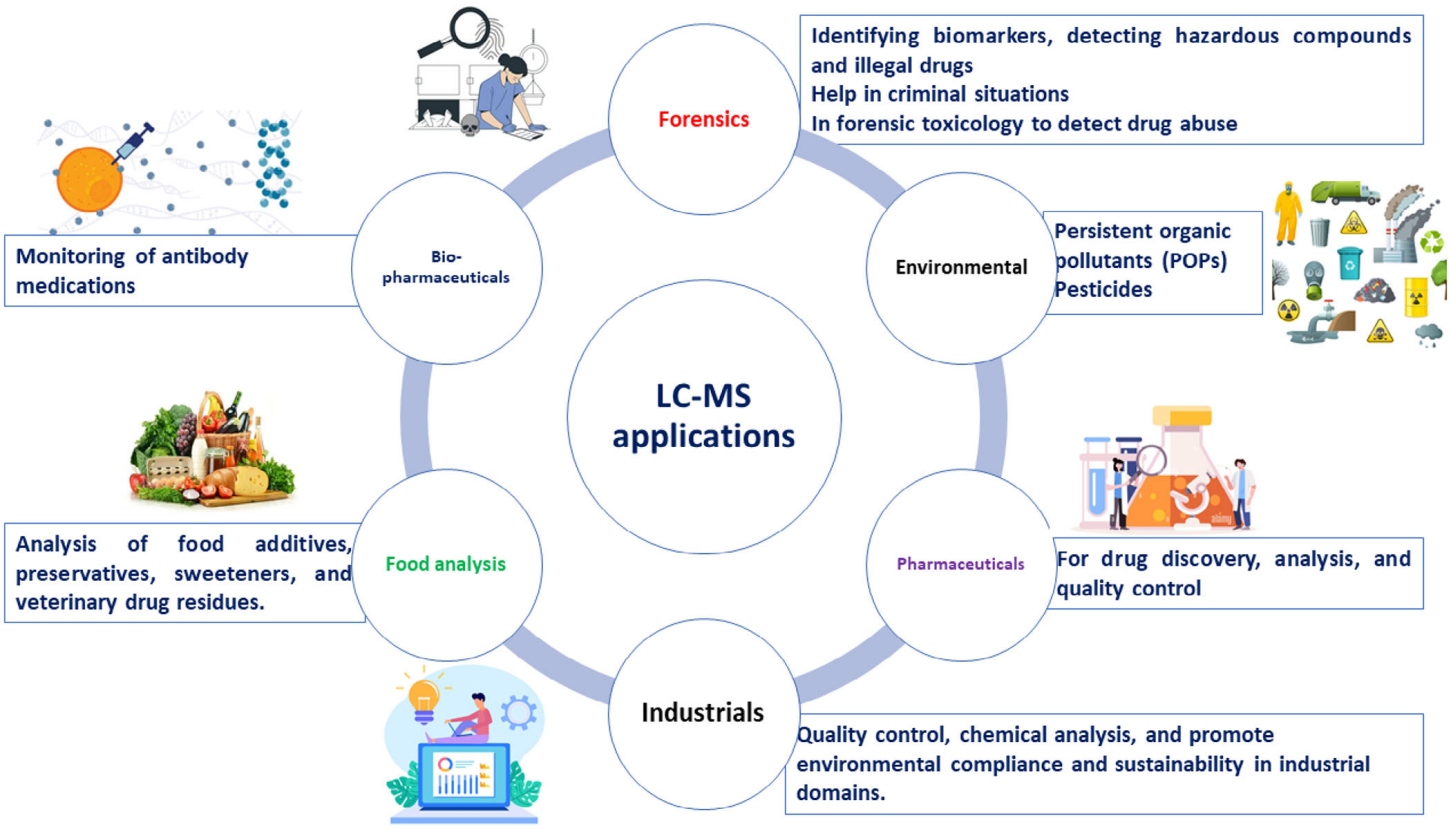
Application of LC–MS in applied and biological fields. LC–MS, liquid chromatography–mass spectrometry.

Contemporary criminal investigations and legal proceedings heavily rely on forensic science [27]. Its significance lies in its capacity to provide impartial, factual evidence for determining guilt or innocence and illuminating the facts surrounding a crime. Due to its precision, sensitivity and adaptability, LC–MS has emerged as a transformative technique in forensic investigation (Figure 3). There is a wide range of applications for LC–MS in forensics [28]. This section provides a general overview of the critical role played by LC–MS in forensic investigation, including identifying biomarkers and detecting hazardous compounds and illegal drugs. It is an essential tool for forensic scientists who try to defend the law and make sense of challenging criminal situations because of its accuracy, sensitivity and adaptability.

It is impossible to stress how crucial forensic analysis is to criminal investigations as it provides a solid scientific framework for evaluating evidence [29]. The LC–MS has shown to be an excellent resource in this situation. Its capacity to accurately separate and identify chemicals is crucial in helping to solve crimes and bring offenders to justice [30]. Recently, LC–MS, LC–MS/MS is increasingly used in the determinations of drug concentrations in biological samples obtained from PK or toxicological studies owing to its versatility for high‐throughput. In clinical and forensic toxicology studies, the analytes which have to be determined are unknown. Therefore, the first step, before quantification, is to screen for and identify any compounds of interest [31]. High‐throughput procedures in analytical toxicology mean that numerous relevant intoxicants can be screened for simultaneously using one single procedure. LC–MS and LC–MS/MS are becoming increasingly routine pieces of apparatus, especially for blood and plasma/serum analysis [31].

Regarding locating drugs, LC–MS is superior to more traditional detection techniques. Due to its incredible sensitivity, it can identify minuscule levels of compounds, which help locate narcotics, poisons or other illegal substances at crime scenes. The use of LC–MS in forensic investigations goes beyond the simple identification of substances; it also helps determine the substances’ source, purity and concentration.

### 5.1 Applications for LC–MS on Drug Abuse

Drug analysis is one of the primary uses for LC–MS in forensics [32]. Fundamental components of forensic toxicology include the identification and measurement of illegal substances [30]. In this area, LC–MS has unmatched capabilities. It can recognize many medicines, including those with identical molecular properties or those found in intricate matrices. Frequently, LC–MS/MS plays a vital role in PKs and pharmacodynamics (PD) studies [33]. In toxicology, LC–MS or MS/MS is so frequently used because first it can be used for non‐volatile and heat‐labile compounds, unlike gas chromatography–mass spectrometry (GC–MS). Another very important and advantageous factor in the use of LC–MS/MS compared to GC–MS is that it is possible to avoid processes of derivatization of the analytes in order to make them volatile and/or analysable by GC [33]. LC–MS/MS, in addition to its common role for analysing lawful drugs and illicit drugs, can also detect metabolites that come from phase I or II metabolisms. This characteristic differs from other instruments and represents the main advantage related to the use of LC–MS/MS in PK and PD studies or for pharmacotoxicological purposes [33]. Merone et al. used LC–MS/MS to develop a rapid screening method to assess more than 739 licit and illicit substances [34]. Drug testing and forensic toxicology both greatly benefit from LC–MS [30]. Whether evaluating compounds found at crime scenes or detecting narcotics in biological samples like blood, urine or hair, LC–MS excels at producing exact findings. As a result, forensic experts may reliably confirm the presence of certain drugs and their amounts, assisting in identifying the role of substances in criminal actions. Its capacity to distinguish between identical compounds ensures this (Figure 1). LC–MS/MS method was developed for the direct analysis of 17 drugs in oral fluids including cocaine, benzoylecgonine (BEG), cocaethylene, Δ‐9‐tetrahydrocannabinol (Δ‐9‐THC), buprenorphine, 6‐acetylmorphine (6AM), morphine, codeine, methadone, 2‐ethylidene‐1,5‐dimethyl‐3,3‐diphenylpyrrolidine (EDDP), amphetamine, methamphetamine, 3,4‐methylenedioxymethamphetamine (MDMA), methylenedioxyamphetamine (MDA), 3,4‐methylenedioxy‐*N*‐ethylamphetamine (MDE), ketamine and *N*‐methyl‐1‐(1,3‐benzodioxol‐5‐yl)‐2‐butanamine (MBDB) [35]. LC–MS method was validated to quantify 30 synthetic cathinones appearing in the illicit market in postmortem blood, including *N*‐ethylpentylone and eutylone [36]. Moreover, a UPLC–MS/MS method was developed for the detection and quantification of 84 drugs and pharmaceuticals, including antipsychotics and antidepressants, some of the most important groups of drugs of abuse: opiates, cocaine, cannabinoids, amphetamines, benzodiazepines and new psychoactive substances in postmortem blood [37]. Additionally, in another study that compared LC–MS–MS and enzyme‐linked immunosorbent assay (ELISA) in the detection of illicit drugs with toxicological impact in urine samples, the results revealed that LC–MS is a sensitive and cost‐effective alternative tool to ELISA screening methods for urine specimens [38]. Examples of applications of LC–MS analysis in drug abuse detection in biological samples were listed in Table 1.

**TABLE 1:** Applications of liquid chromatography–mass spectrometry (LC–MS) analysis in drug abuse and fluid analysis.

Sample type	Drug	LC–MS interface/MS analyser	Refs.
Dried blood spot	Carbamazepine, lamotrigine, valproic acid	ESI+/Triple quad MS	[46]
Dried blood spot	Fluoxetine, norfluoxetine	ESI+/Triple quad MS	[47]
Plasma	Buprenorphine, norbuprenorphine, naloxone	ESI+/5500 Triple quad MS	[48]
Human plasma, human urine	12 metabolites	ESI+/5500 Triple quad MS	[49]
Postmortem human blood	30 cathinones	Jet stream electrospray ionization+/Triple quad MS	[36]
Postmortem blood	84 drugs of abuse and pharmaceuticals	ESI+/−/TQD	[37]
Human urine	88 drugs and illicit drugs	ESI+/Triple quad MS	[38]
Postmortem human blood, postmortem muscle tissue	29 drugs and metabolites	ESI+/Triple quad, tandem MS	[45]
Oral fluid	17 drugs of abuse	ESI+/Triple quad MS	[35]

Recently, the use of LC–MS in doping control has become an essential tool in modern sports analytics. LC–MS is now used as a powerful tool for the detection of performance‐enhancing drug, their metabolites and masking agents in biological matrices such as urine, blood and hair [39]. Recent advancements in HRMS and LC–MS/MS have significantly improved doping detection capabilities by lowering detection limits and enabling metabolite profiling [40]. LC–MS offers high sensitivity, selectivity and the ability to analyse a wide range of prohibited substances [41], including anabolic steroids (testosterone and nandrolone), stimulants (amphetamines and ephedrine), peptide hormones (erythropoietin and human growth hormone), β2‐agonists (clenbuterol and salbutamol) and diuretics or masking agents (furosemide and probenecid) [42].

### 5.2 Applications for LC–MS on Blood, Oral and Urine Sample Analysis

LC–MS is crucial for evaluating blood and urine samples in forensic and therapeutic contexts and its usefulness in drug detection [43]. Biomarker studies are necessary for comprehending physiological states, diagnosing illnesses and determining exposure to toxins or illicit substances [44]. Forensic pathologists and toxicologists depend on LC–MS's capacity to recognize metabolites and poisons in body fluids [43]. It enables the identification of specific molecules connected to drug use, toxicity or illness. Drug or toxin metabolites are crucial indicators in forensic investigations because they can persist in the body long after metabolizing the parent chemicals. Additionally, poisons that cannot be detected by conventional testing methods can be analysed using LC–MS. Hansen et al. have developed and validated a UPLC–MS/MS method for the quantification of 29 drugs and metabolites with toxicological effects in postmortem blood and muscle tissue [45]. A fast LC–MS/MS method was developed for the quantification of carbamazepine, lamotrigine and valproic acid based on dried blood spot sampling and revealed linearity in therapeutically relevant concentration ranges and compatible with unknown volume sampling and expected hematocrit range of the patient group [46]. Moreover, LC–MS analysis method was developed and validated by da Silva et al. to a simultaneous determination of the antidepressant's fluoxetine and norfluoxetine in dried blood spots revealing linearity range 10–750 ng mL^−1^ and accuracy in the range of 97.98%–110.44% and 100.25%–105.8% [47]. Liu et al. developed and validated a simple, sensitive and rapid LC–ESI–MS/MS method for a simultaneous quantification of naloxone, buprenorphine and its metabolite norbuprenorphine in human plasma [48]. Moreover, blood and urine samples were used for a simultaneous detection and quantification of 12 metabolites (sulfocysteine, guanidinoacetate, creatine, pipecolic acid, Δ^1^‐piperideine‐6‐carboxylate [P6C], proline, Δ^1^‐pyrroline‐5‐carboxylate [P5C], and the B6‐vitamers) using LC–MS/MS method for the differential diagnosis of inherited metabolic epilepsies [49].

## 6 Applications for LC–MS in Environmental Sciences

This subsection explores the crucial function of LC–MS in environmental risk assessment. Persistent organic pollutants (POPs) concentrations in environmental samples are precisely measured by LC–MS, which helps in assessing the potential risk that these pollutants pose to ecosystems and human populations (Table 2). These analyses are critical in influencing policy choices, directing pollution prevention efforts and encouraging environment‐friendly activities. Environmental evaluation and monitoring are crucial components of ethical resource management. LC–MS has gained increased recognition in this field due to its ability to provide accurate and reliable analytical data across several environmental matrices [50]. Scientists and environmentalists may detect and quantify a range of substances using LC–MS to understand environmental changes and their effects better. LC–MS is crucial in guaranteeing the security and quality of essential resources [51]. The health of both people and the environment is significantly impacted by the quality of soil and water, which are crucial elements of the Earth's ecosystems. It can detect contaminants and pollutants, from organic molecules to heavy metals, even at low levels. LC–MS's sensitivity and specificity are essential for precisely identifying and measuring these compounds [52]. Additionally, by thoroughly assessing pollutants in water and soil samples, LC–MS supports environmental impact assessments [53]. Making educated judgments on land use, pollution mitigation, and cleanup techniques requires this knowledge. By providing knowledge on the causes and degree of contamination, LC–MS aids in the creation of practical solutions to restore and safeguard our natural habitats. Chemicals known as POPs pose severe threats to ecosystems and public health because they are resistant to degradation and continue to exist in the environment. Pesticides, polychlorinated biphenyls (PCBs) and dioxins are among the POPs that may be easily identified and measured using LC–MS [54]. LC–MS/MS method was developed and validated for the determination of 21 antibacterial substances in natural and organic liquid fertilizers by the analysis of 62 samples of natural and organic liquid fertilizers, showing that over 24% of the tested samples were contaminated with antibiotics, mainly from the group of tetracyclines and fluoroquinolones [55]. LC–MS/MS technique was used as a simple and sensitive analytical method for the simultaneous determination of 311 active substances of pesticides in agricultural soils using and GC–MS techniques [56]. UHPLC–MS/MS analytical method was developed for the identification of 52 pharmaceutical products and to monitor their presence in drinking water [57]. LC–MS/MS was used for the determination of fungicide residues in soil and detection of its environmental risk assessment [58]. LC–MS has transformed environmental sciences by enabling pollutant detection, analysis and management in environmental matrices. In order to safeguard the ecosystems on our planet and ensure a sustainable future, its applications in the study of soil and water, as well as in the identification and quantification of POPs, have become essential.

**TABLE 2:** Applications for liquid chromatography–mass spectrometry (LC–MS) in environmental sciences and food safety.

Application	Substance	Matrix	Methodology	Results	Refs.
Environmental monitoring	POPs	Water, soil, air	LC–MS, LC–MS/MS	Detection of low concentrations of POPs such as pesticides, PCBs, and dioxins	[50, 54]
Antibacterial residues in fertilizers	Antibiotics (tetracyclines, fluoroquinolones)	Organic and natural liquid fertilizers	LC–MS/MS	Over 24% of samples contaminated with antibiotics	[55]
Pesticide residues in agricultural soil	Pesticides	Agricultural soils	LC–MS/MS, GC–MS	Simultaneous determination of 311 pesticide residues	[56]
Pharmaceutical contamination in water	Pharmaceutical products (e.g. painkillers)	Drinking water	UHPLC–MS/MS	Detection of 52 pharmaceutical products in drinking water	[57]
Fungicide residues in soil	Fungicides	Soil	LC–MS/MS	Determination of fungicide residues and assessment of environmental risks	[58]
Food safety	Caffeine	Beverages (Iced tea, coffee, energy drinks)	LC–MS/MS	Detection limits: 0.04 mg L^−1^ for caffeine in various beverages	[62]
Herbicide residue in beverages	Diquat	Water and beverages (e.g. iced tea, soft drinks)	LC–MS/MS	Recovery: 85.9%–115%, RSD: 3%–8%	[63]
Food additives	Plasticizers (e.g. phthalates, sebacates)	Food products	LC–MS/MS	Detection of 60 additives with low limits of quantification in the µg kg^−1^ range	[64]
Polyethylene terephthalate (PET) film characterization	Virgin vs. recycled PET films	Food packaging materials	LC–MS	Differentiation of virgin and recycled PET materials	[65]
Veterinary drug residues	Antibiotics, anti‐inflammatory agents, tetracyclines	Milk, meat, eggs, baby foods, infant formula, etc.	LC–MS/MS	Detection of 152 veterinary drug residues with ≥94% probability of detection	[67]
Porcine gelatin markers	Porcine gelatin	Confectionery products	LC–MS/MS	Detection limit: 0.09% to	

Abbreviations: PCBs, polychlorinated biphenyls; POPs, Persistent organic pollutants; RSD, relative standard deviation; UHPLC–MS, ultra‐high‐performance liquid chromatography–mass spectrometry.

## 7 Applications of LC–MS in Analysis of Food Additives and Beverages

LC–MS contributes to regulatory compliance, consumer protection and overall food and beverage industry improvement through these applications (Table 2). Ensuring the safety and quality of food and beverages is paramount for both consumers and regulatory agencies [2]. LC–MS has emerged as a powerful analytical tool for detecting, identifying, and quantifying food additives, preservatives, sweeteners and veterinary drug residues in various food matrices [59]. Through these applications, LC–MS contributes significantly to regulatory enforcement, consumer protection and overall advancements in the food and beverage industry. One of the critical applications of LC–MS in the food sector is the analysis of food additives and preservatives. LC–MS provides high sensitivity and specificity in detecting synthetic additives, antioxidants and stabilizers, ensuring compliance with regulatory limits [60]. LC–MS enables precisely identifying and quantifying these substances, ensuring that their usage in food products complies with regulatory limits [61]. Additionally, LC–MS plays a pivotal role in detecting food fraud and adulteration, safeguarding consumer trust and product integrity [61]. LC–MS/MS method was developed to assess the caffeine levels in soft beverages (iced tea, ice coffee and energy drinks) available in Istanbul, Turkey with a limit of quantification and a limit of detection values for caffeine analysis that was 0.13 and 0.04 mg L^−1^, respectively [62]. A new simple, fast and sensitive LC–MS/MS method was developed for the determination of diquat in water and beverages with an average recovery ranging from 85.9% to115% and associated relative standard deviation (RSD %) within the range 3–8 [63]. LC–MS/MS technique was used for the determination of 60 food additives mainly plasticizer, including 28 phthalates and 32 further compounds such as sebacates, adipates, citrates and fatty acid amides, revealing good reproducibility and high precision with limits of quantification in the low µg kg^−1^ food range [64]. Recently, LC–MS analysis was used for the chemical characterization of virgin and recycled polyethylene terephthalate films in food and beverages [65]. The use of veterinary medications in animal husbandry brings up concerning drug residues in foods obtained from animals [66]. In order to make sure that animal products entering the food supply chain are safe for human consumption, LC–MS provides a dependable method for monitoring and measuring these residues [66]. The food business can prove compliance with regulatory requirements and, more crucially, reassure customers of the safety of the food items that they buy by using LC–MS methodologies. LC–MS is critical to upholding food and beverages’ quality, safety and integrity. LC–MS/MS method was optimized to screen 152 veterinary drug residues, including 105 antibiotic residues, anti‐inflammatory and antiparasitic agents, 23 beta‐lactams, 14 aminoglycosides and 10 tetracyclines in a wide variety of food commodities, including milk‐based ingredients and related products (e.g., milk fractions, infant formula, infant cereals and baby foods), meat‐ and fish‐based ingredients and related products (fresh, powdered, cooked, infant cereals and baby foods), and other ingredients based on eggs, animal fat and animal byproducts with probability of detection at the STC ≥94% [67]. Moreover, LC–MS/MS was used for the identification of porcine gelatin markers in confectionery products with detection limit ranging from 0.09% to 0.89% [68].

## 8 Applications for LC–MS in Biopharmaceuticals

Monoclonal antibodies are pivotal in treating infectious diseases, autoimmune ailments and cancer [69]. LC–MS presents a sophisticated analytical arsenal for this domain. Leveraging the LC–MS framework alongside MS empowers the comprehensive assessment of antibody medications [70]. It facilitates the detection of post‐translational modifications, such as glycosylation and phosphorylation, which can significantly impact drug efficacy and safety [70]. In biological pharmaceuticals, ensuring that the purity and integrity of antibody drugs is paramount, and LC–MS plays a pivotal role [71]. It furnishes quantitative and qualitative insights into the constituents of antibody drugs, thereby facilitating compliance with regulatory mandates and the dependable production of top‐tier therapeutics [72]. Glycoproteins, a specialized category of biopharmaceuticals, present distinctive analytical complexities due to their diverse glycan structures [73, 74]. LC–MS emerges as the go‐to choose for glycoprotein scrutiny, enabling the precise identification and quantification of glycan components—an imperative aspect for comprehending these biologics’ therapeutic potential and immunogenicity [75]. Additionally, LC–MS‐guided glycan profiling contributes to the development of safer and more efficacious biotherapeutics [76]. UHPLC–MS/MS method was developed for a fast determination of immunogenic synthetic peptide in a conjugate with bovine serum albumin as a carrier protein formulated with a vaccine adjuvant [77]. Native LC–MS has been reported for a rapid assessment of molecular mass and deep characterization of the heterogeneity of complex, recombinantly produced therapeutic proteins [78]. Recent advances in LC–MS‐based characterization of protein‐based biotherapeutics mastering were reviewed by Graf et al. [79]. By scrutinizing glycan profiles across various production batches or conditions, researchers can fine‐tune glycosylation patterns in biopharmaceuticals, ensuring consistent product quality and mitigating the risk of adverse reactions [76]. LC–MS has transformed biopharmaceuticals, offering exacting and adaptable tools for analysing antibody drugs, glycoproteins and other protein‐based therapeutics. Its role in quality assurance, structural elucidation and optimization of biological drugs is pivotal in advancing innovative and efficacious treatments (Figure 3).

## 9 Applications of LC–MS in Pharmaceuticals

The pharmaceutical sector significantly relies on LC–MS for drug discovery, analysis and quality control to assure the safety and effectiveness of medicines. This section examines the crucial function of LC–MS in this field, including pharmaceutical quality assurance and regulatory compliance. As lipids play vital roles in biological systems, lipidomics, a fast‐developing field, is essential in drug discovery [80]. As a result of the complete lipid profile made possible by LC–MS, lipid‐related disorders and therapeutic development are better understood [81]. This section explores LC–MS's uses in lipidomics while stressing its value for pharmaceutical research. The measurement of phospholipids is crucial for pharmaceutical research as they are critical parts of cell membranes [82]. LC–MS offers sensitive and precise techniques for measuring phospholipids to formulate lipid‐based drugs and comprehend how phospholipids affect drug delivery [82].

Enantiomers of chiral medications frequently occur and have various pharmacological properties [83]. Enantiomers are separated and quantified using LC–MS, facilitating the creation of safer and more potent medications. The function of chiral drug analysis and the significance of LC–MS in drug development are covered in this section. The creation of effective chiral LC–MS techniques is a difficult challenge [51]. Chiral LC–MS is important to the study and development of pharmaceuticals. An enantioselective method was developed for quantifying amphetamine‐type chiral illicit drugs (CIDs) in wastewater and surface water using solid‐phase extraction and quantified via LC–MS/MS [84]. A chiral LC–MS/MS method was developed and validated for the separation and quantification of four synthetic cathinones in human whole blood as differentiation of their (*R*)‐ and (*S*)‐enantiomers is relevant in clinical and forensic toxicology [85].

Pharmaceutical development is crucially dependent on PKs and bioavailability. To study drug absorption, distribution, metabolism and excretion (ADME), LC–MS analyses the concentration of drugs and metabolites in biological matrices [86]. This section emphasizes the use of LC–MS in bioavailability and PK research. LC–MS is essential for determining drug levels in patient samples in both preclinical and clinical trials [87]. By enabling drug efficacy and safety evaluation, it aids in the decision‐making process for medication development. This subsection examines the many applications of LC–MS in the clinical phases of pharmaceutical research. The assessment of PKs and bioavailability of D4‐cystine was employed by LC–MS/MS to generate calibration curves for the determination of levels of D4‐cystine and endogenous cystine in mice [88]. LC–MS/MS method was developed and validated for the quantification of 5‐Amino‐1‐methyl quinolinium in rat plasma and urine samples to test its PK and oral bioavailability [89].

## 10 Innovations in LC–MS

Recent developments in LC–MS technology have significantly expanded its application potential, opening new avenues in various scientific domains. This section explores the latest innovations and emerging trends in LC–MS, highlighting its transformative potential and future prospects. LC–MS has evolved considerably in response to the ever‐changing landscape of analytical instrumentation, with recent advancements elevating its performance and broadening its range of applications. Recent developments have elevated LC–MS to new levels and improved its adaptability and capabilities. Recently, several hyphenated techniques were developed by combining IMS and nuclear magnetic resonance spectroscopy which are complementary analytical methods integrated with LC–MS [7]. This contact improves analytical skills and widens the range of possible applications. HRMS equipment has changed LC–MS by improving mass accuracy and sensitivity [90]. HRMS enabled the identification of substances with higher certainty and to detect and quantify them at lower quantities [91]. Although TOF mass analysers have a lower mass resolution than Fourier transform (FT) mass analysers (FTICR), they virtually have no upper *m*/z limit and are thus particularly useful to identify singly charged ions of high molecular weight. Their fast response/scan rate is also advantageous for applications requiring short acquisition times, for example in UPLC‐ and LC–HRMS analysis. Orbitrap and TOF instruments typically produce a mass accuracy of 2–5 ppm, which is sufficient to assign molecular formulae to candidate molecules [33]. However, the mass accuracy of TOF analysers can vary depending on the specific variant used. For example, reflectron TOF generally offers higher mass accuracy due to its extended ion flight path and improved energy focusing, whereas linear TOF tends to have lower mass accuracy. Q‐TOF instruments, which combine quadrupole and TOF capabilities, provide enhanced resolution and accuracy compared to standard TOF configurations. However, it becomes more difficult to identify a compound as the *m*/*z* of the ion increases, as more possible elemental compositions will fall within a certain mass error, making the elemental composition assignment more difficult. Although HRMS was originally used in drug metabolism and metabolite identification studies due to its superior qualitative performance, it is nowadays increasingly used to perform reliable and sensitive quantitative analyses [92]. The sensitivity of HRMS is currently often comparable or superior to that of traditional QQQ instruments, due to the possibility to construct EICs with narrow mass extraction windows. This was, for example, demonstrated for the analysis of protease inhibitors, tyrosine kinase inhibitors, steroids and metanephrines in plasma samples comparing a new Orbitrap with a recent QQQ [93]. LC–MS systems have become more compact and portable, making them accessible for on‐site analysis and fieldwork. It is particularly beneficial in applications such as environmental monitoring and food safety [2]. Automation and advanced data processing techniques have optimized workflows for the LC–MS. Rapid data interpretation and predictive analysis are made possible by artificial intelligence (AI) and ML [2]. The interpretation of LC–MS‐driven data is a significant issue, particularly given the variety of metabolite classes identified by LC–MS, specially in the plant kingdom, in contrast to the automated GC–MS analytical workflow, which has been refined for decades [2]. When utilized appropriately, a number of computational methods that have been developed to improve the annotation of the observed metabolites with varying degrees of certainty could be of great use [2].

Recently, IMS and data‐independent acquisition approaches have been used to support LC–MS advancement by enhancing the separation capacity and reproducibility of complex biological analyses. The utility of high‐resolution IMS in untargeted metabolomics was demonstrated to enhance confidence in metabolite annotation and isomer differentiation [94]. Among data‐independent acquisition approaches, sequential window acquisition of all theoretical mass spectra (SWATH‐MS) provide a systematic and unbiased acquisition of all detectable precursor ions, enabling comprehensive and reproducible quantitation of analytes in complex matrices [95]. The work done by Gillet et al. demonstrated a SWATH‐MS as a powerful tool to improve biomarker discovery and enhance large‐scale clinical proteomics [95]. The dual application of IMS with data‐independent acquisition can offer deeper insights into complex biological systems with greater analytical confidence in LC–MS workflows.

Moreover, the application of AI and ML is rapidly applied to LC–MS data analysis for complex datasets processing and interpretation. ML can reduce dimensionality, improve biomarker discovery and support robust statistical modelling in metabolomics studies [96, 97, 98]. Moreover, deep learning is emerged to improve feature detection accuracy in untargeted metabolomics workflows [99]. As LC–MS datasets continue to grow in complexity and scale, AI‐driven approaches offer promising solutions for data integration, automation and improved analytical throughput.

## 11 Summary and Outlook

The significance of LC–MS in biology and life sciences cannot be overstated. With its unparalleled precision, sensitivity and versatility, this technology has revolutionized both academic research and professional applications. In the years ahead, LC–MS is poised to further transform these fields, playing a crucial role in addressing emerging challenges such as pharmaceutical innovation and environmental sustainability. As LC–MS technology continues to advance, it will unlock new frontiers for scientific discovery and commercial applications, empowering researchers to push the boundaries of human knowledge. Its exceptional reliability, exquisite sensitivity and remarkable adaptability have cemented its status as an indispensable analytical tool across diverse domains. With each breakthrough, LC–MS reaffirms its pivotal role in driving innovation. As technology matures and novel applications emerge, it will remain at the forefront of scientific inquiry, fostering groundbreaking discoveries that benefit society. The biological sciences have already witnessed the profound impact of LC–MS, and its potential for the future is boundless.

## Conflicts of Interest

The author declares no conflicts of interest.

## Data Availability

The author has nothing to report.
